# Antimalarial Activity of* Croton macrostachyus* Stem Bark Extracts against* Plasmodium berghei In Vivo*

**DOI:** 10.1155/2018/2393854

**Published:** 2018-06-10

**Authors:** Jackie K. Obey, Moses M. Ngeiywa, Paul Kiprono, Sabah Omar, Atte von Wright, Jussi Kauhanen, Carina Tikkanen-Kaukanen

**Affiliations:** ^1^University of Eastern Africa, Baraton, School of Health Sciences, Department of Medical Laboratory Sciences, P.O. Box 2500, 30100 Eldoret, Kenya; ^2^University of Eldoret, Department of Biological Sciences, P.O. Box 1125, 30100 Eldoret, Kenya; ^3^University of Eldoret, Department of Chemistry, P.O. Box 1125, 30100 Eldoret, Kenya; ^4^Kenya Medical Research Institute, Center of Biotechnology and Research Development, Department of Malaria, 00200 Nairobi, Kenya; ^5^University of Eastern Finland, Institute of Public Health and Clinical Nutrition, Kuopio, Finland; ^6^University of Helsinki, Ruralia Institute, 50100 Mikkeli, Finland

## Abstract

There is an increasing need for innovative drug and prophylaxis discovery against malaria. The aim of the present study was to test* in vivo* antiplasmodial activity of* Croton macrostachyus *H. (Euphorbiaceae) stem bark extracts from Kenyan folkloric medicine. Inbred Balb/c mice were inoculated with erythrocytes parasitized with* Plasmodium berghei *(ANKA). Different doses (500, 250, and 100 mg/kg) of* C. macrostachyus* ethyl acetate, methanol, aqueous, and isobutanol extracts were administrated either after inoculation (Peters' 4-day suppressive test) or before inoculation (chemoprotective test) of the parasitized erythrocytes. All the extracts showed significant suppression of parasitemia compared to control (*p* < 0.001): for the ethyl acetate extract in the range of 58–82%, for the methanol extract in the range of 27–68%, for the aqueous extract in the range of 24–72%, and for the isobutanol extract in the range of 61–80%. Chemoprotective effect was significant (*p* < 0.001) and the suppression caused by the ethyl acetate extract was between 74 and 100%, by the methanol extract between 57 and 83%, and by the isobutanol extract between 86–92%. The study showed that it is possible to inhibit the growth of the parasites by various stem bark extracts of* C. macrostachyus* in Balb/c mice supporting the folkloric use of the plant against malaria.

## 1. Introduction

 Medicinal plants have been used to cure parasitic infections from time immemorial. About 40% of the modern drugs and approximately 75% of drugs for infectious diseases are of natural origin. The number of drug-like molecules possibly present in the vast amount of species (fungi, bacteria, marine invertebrates, and insects) has been estimated to exceed 10^60^ [1]. As a source of novel drugs, plants remain grossly understudied and underused, especially in the developed world [2, 3]. A world health organization study has shown that 80% of the world's population relies solely upon medicinal plants as a source of remedies for the treatment of diseases [2]. In China, India, Africa, and Latin America, modern drugs are not available, or, if they are, they often prove to be too expensive, unavailable, or inaccessible.

Malaria has historically been among the most deadly parasitic infections in many tropical and subtropical regions [4, 5].* Plasmodium falciparum* is the most important agent of human malaria, transmitted by the* Anopheles* mosquito into the human blood. Worldwide 212 million malaria cases were reported in 2015. Between 2010 and 2015 the new incident malaria cases have decreased by 21% and mortality due to malaria has decreased by 29%. Until recently, malaria used to be the leading cause of death among children in sub-Saharan Africa [5]. The improvements can be attributed to WHO-recommended core interventions, vector control, chemoprevention, diagnostic testing, and treatment, which all have proven to be cost-effective.

Emerging parasite resistance to antimalarial medicines as well as mosquito resistance to insecticides could render some of the current tools ineffective and trigger a new rise in global malaria mortality. The resistance of* P. falciparum* to 4-aminoquinolines (chloroquine, amodiaquine), antifols, and dihydrofolate reductase inhibitors (pyrimethamine, proguanil) in many endemic regions [6] and the emergence of* in vitro* and* in vivo* resistance to aminoalcohols (quinine, mefloquine, and halofantrine) have been reported in some areas of Southeast Asia [7–9]. Except antifolate antimalarial drugs other commonly used antimalarial agents are based on plant-derived compounds, quinine, and artemisinin derivatives, which remain vital drugs in the treatment of malaria [10, 11].

*Croton macrostachyus* Hochst. ex Delile is a species of the genus* Croton* L., Euphorbiaceae family, commonly known as the spurge family.* C. macrostachyus* is regarded as a multipurpose tree by subsistence farmers in Ethiopia, Kenya, and Tanzania and the species has potential in playing an important role in the primary healthcare. The bark, fruits, leaves, roots, and seeds of* C. macrostachyus* are reported to possess diverse medicinal properties and* C. macrostachyus* is used as herbal medicine for at least 61 human and 20 animal diseases and ailments [12]. In the distribution area there is a high degree of medicinal use consensus for bleeding, blood clotting, cancer, constipation, diarrhea, epilepsy, malaria, pneumonia, purgative, ringworm, skin diseases or infections, stomach ache, typhoid, worm expulsion, and wounds [13]. Leaf decoction, infusion, or maceration, stem or root bark, and leaf sap of* C. macrostachyus* are taken as a purgative and vermifuge, and the seed oil is a very powerful purgative [12].* C. macrostachyus* is also used for medicinal purposes in combination with other plant species [12, 13]. Members of the genus* Croton* and different parts of plant have been used traditionally to treat infectious diseases such as measles and typhoid fever in Kenya [14] and against malaria in Ethiopia [15]. Antimalarial activity against* Plasmodium berghei* in mice has been found from* C. macrostachyus* leaves [16, 17] and from fruit and root [18]. In the present study antiplasmodial activity of* C. macrostachyus *stem bark extracts was investigated in a rodent model against* P. berghei.*

## 2. Materials and Methods

### 2.1. Plant Collection and Extraction

*C. macrostachyus* stem bark was collected from the nature preserve of the University of Eastern Africa, Baraton community in Nandi District of Kenya. Baraton is located 10 km from Kapsabet, the headquarters of Nandi Central District. A voucher specimen was deposited in the herbarium at the Department of Biological Sciences, University of Eastern Africa, Baraton, and the National Museums of Kenya for identification.

### 2.2. Preparation of the* C. macrostachyus* Extracts

The stem bark extracts were prepared as described before [19]. Shortly, the fresh stem bark was cut into small pieces using a pen knife. The cut bark was air-dried in a shaded area for three weeks. The air-dried bark was powdered using a mechanized hand grinder. The powdered material (500 g) was soaked into ethyl acetate, absolute methanol, distilled water, or isobutanol for 24 hours. The soaked extract was separated from the plant residue by using a Buchner funnel. The extract was separated from the solvent in a rotary evaporator device (Rotavapor R300) at 40°C. Each plant extract was then dissolved in dimethyl sulfoxide (DMSO, Rankem, India) to the concentration of 500 mg/ml.

### 2.3. Preparation of Parasites

*Plasmodium berghei* strain ANKA, a chloroquine-sensitive strain of malaria parasite, was used in the* in vivo* study. The parasites were maintained by injecting serial passages of infected blood intraperitoneally (ip) into a Balb/c mouse and were then collected by cardiac puncture. The percent parasitemia and the erythrocytes were counted using the white blood cell count method. The blood was then diluted with isotonic saline to obtain 2 × 10^7^ parasitized erythrocytes/ml. The test animals were infected intraperitoneally with 0.2 ml of the parasitized erythrocytes.

### 2.4. Acute Toxicity Experiment

An acute toxicity test was carried out to ascertain the safety of the extract. Six groups of six clean uninfected BALB/c mice were used in the toxicity test. For the test groups an oral dose of 0.2 ml was given per animal consisting of either 500 mg/kg, 250 mg/kg, or 100 mg/kg of the extract according to the body weight. The negative control group received 0.2 ml of 10% Tween per animal and the positive control group received artemether. The weights of all animals were taken before and after the experiment. The animals in each group were observed for any change in physical activity and signs of abnormal growth or disease condition. This included observations of mortality, hair erection, tremors, lacrimation, convulsions, salivation, diarrhea, and abnormal features in organs and blood.

### 2.5. Peters' 4-Day Suppressive Test

The parasites used for this study were obtained from the Kenya Government Medical Research Institute (KEMRI), Center for Biotechnology and Research Development (CBRD), Department of Malaria. The study design was a quantitative case control study described by Peters in 1975 [20]. Infected mice groups were treated with various extract concentrations and positive control group was treated with artemether (PC) and negative control group with 10% w/v Tween 80 (NC). Consent to use the experimental animals in the study was obtained from the ethical committee of the Kenya Medical Research Institute, Center of Biotechnology and Research Development, Department of Malaria. Male 6–8 weeks old BALB/c mice weighing 20 ± 2 g were selected for the* in vivo* study. Each mouse was infected intraperitoneally by injecting 0.2 ml of 2 × 10^7^ erythrocytes/ml parasitized with* Plasmodium berghei* strain ANKA. Groups of six mice were delivered orally with either crude ethyl acetate, isobutanol, methanol, or aqueous extracts using doses of 500, 250, and 100 mg/kg body weight. Each study group included PC and NC groups. The plant fractions were dissolved in 10% w/v Tween 80 with the aid of ultrasonication and diluted with distilled water to the test concentrations. On days 0, 1, 2, and 3 the animals were treated once orally with the different doses of the extracts in a volume of 0.02 ml/g body weight. The mice received NAFAG pellets (9009 PAB – 45) (Nafag AG, Switzerland) as a diet and were held at room temperature. The survival of the mice in all the groups was checked twice a day. Parasitized erythrocytes (RBC) were counted in Giemsa stained thin films from tail blood on day 4. The average parasitemia was calculated as

(1) % Parasitemia=Number  of  parasitized  RBCTotal  number  of  RBC×100.

The percentage suppression of parasitemia (PSP) for each plant extract was calculated as [21]

(2) Suppression %=Parasitemia  in  negative  control−Parasitemia  in  study  groupParasitemia  in  negative  control×100.

### 2.6. Chemoprotective Activity

The chemoprotective prophylaxis* in vivo* study was set up and carried out by first treating the animals for four days with the different doses of the studied extracts before exposing them to infection. BALB/c male mice of 6–8 weeks old weighing 20 ± 2 g were selected for the chemoprotective study. For each extract, 6 animals were selected as positive controls and negative controls and for test animals. Three different doses of the prepared extracts of 500, 250, and 100 mg/kg body weight were administrated orally. Each individual in a group of thirty BALB/c mice was infected by injecting 0.2 ml of 2 × 10^7^ parasitized erythrocytes/ml intraperitoneally. The mice received NAFAG pellets (9009 PAB-45) as a diet and were held at room temperature. The survival of the mice in all groups was recorded twice a day. Parasitized erythrocytes were counted in Giemsa stained thin films prepared from tail blood on day 4. The percentage suppression of parasitemia for each plant extract was calculated.

### 2.7. Statistical Analysis

Data are expressed as the mean ± the standard error of the mean. One-way analysis of variance (ANOVA) followed by Tukey's honest significant difference post hoc test was used to determine statistical significance in the comparisons of parasitemia suppression. Values of *p* < 0.001 were considered statistically significant.

## 3. Results

### 3.1. Acute Toxicity Experiment

The results from the toxicity experiment showed that all animals in the ethyl acetate, methanol, and water extract groups were normal during the observations and at the end of the study period. The mice of the isobutanol extract treatment group showed signs of acute toxicity. They showed signs of tremor on the third day. On a closer observation, the white fur seemed thin and slight and was erected. None of the animals experienced salivation, lacrimation, diarrhea, or convulsions. Two of the six mice in the isobutanol dose group of 500 mg/ml died before the end of the treatment period.

### 3.2. *In Vivo* Peters' 4-Day Suppressive Test

The parasitemia in the negative control group was significantly higher than in any of the treatment groups (*p* < 0.001) (Table 1). All the animals in the positive control group displayed suppression of parasitemia of 88–100% (Table 2). The ethyl acetate extract induced 82%, 58%, and 62% suppression, the methanol extract induced 68%, 34%, and 27%, and the aqueous extract 72%, 44%, and 24% suppression for the doses of 500, 250, and 100 mg/kg, respectively. For the doses of 500, 250, and 100 mg/kg the isobutanol extract induced the suppression of 80%, 61%, and 73%, respectively (Table 2).

### 3.3. Chemoprotective Activity

In the chemoprotective assay the parasitemia in the negative control group was significantly higher than in any of the test group (*p* < 0.001) (Table 3). All the animals in the positive control group displayed suppression of parasitemia of 99% (Table 4). In the ethyl acetate extract group suppression of parasitemia was 100%, 93%, and 74%, in the methanol extract group 83%, 65%, and 57%, and in the isobutanol extract group 92%, 84%, and 86% for the doses of 500, 250, and 100 mg/kg, respectively (Table 4).

## 4. Discussion

Despite the overall favorable development in global malaria incidence and mortality rates, malaria still remains one of the gravest public health threats to human life in many regions. Furthermore, the consequences of nonfatal malaria episodes pose a major economic burden to working age populations and local communities, especially in many African countries [5]. The current cost-effective options available in the prophylaxis and treatment of malaria are, regardless of their merits, widely considered insufficient to control malaria more efficiently. There is a need for development of new agents owing to the increasing resistance of the parasite to available agents [17].

*C. macrostachyus* leaves [16], crude leaf extract and chloroform fractions [17], and crude 80% methanol extracts of the fruit and root [18] have been shown to possess antimalarial activity against* Plasmodium berghei* in mice. We have recently shown antimicrobial activity of* Croton macrostachyus *H. (Euphorbiaceae) stem bark extracts against several human pathogenic bacteria and a fungus [19]. In the present study different crude stem bark extracts from* C. macrostachyus* induced a decrease in parasite density* in vivo*.* In vivo* antiplasmodial activity can be classified as moderate, good, and very good if an extract displayed percentage parasitemia suppression equal to or greater than 50% at a dose of 500, 250, and 100 mg/kg body weight per day, respectively [22]. In the present study very good impact was achieved with ethyl acetate (62%) and isobutanol (73%) extracts (over 50% with 100 mg/kg) in the Peters' 4-day suppressive test. Methanol (68%) and water (72%) extracts had moderate activity (over 50% with 500 mg/kg of the extracts). In the chemoprotective assay ethyl acetate (74%), methanol (57%), and isobutanol extracts (86%) had very good impact (over 50% with 100 mg/kg of the extract).

The activity of* C. macrostachyus* stem bark extracts is comparable to studies where antiplasmodial activity has been related to a range of several classes of secondary plant metabolites including alkaloids and sesquiterpenes, triterpenes, flavonoids, inonoids, and quassinoids [23]. These mostly amphiphile compounds have been described to protect erythrocytes against hypotonic hemolysis [24]. Here we found that the highest percent for the suppression of parasitemia was obtained with the dose of 500 mg/kg of* C. macrostachyus* stem bark ethyl acetate extract (100% in chemoprotective and 82% in Peters' 4-day suppressive test). Very good impact (100 mg/kg) was achieved with ethyl acetate extract in the Peters' 4-day suppressive test (62%) and in the chemoprotective assay (74%). The main components of* C. macrostachyus* stem bark are lupeol, betulin, and fatty acids [13]. Because of their solubility properties one could conclude that the isobutanol and methanol extracts contained mixtures of these compounds. According to our previous work lupeol is extracted from* C. macrostachyus* stem bark by ethyl acetate [19]. Lupeol is a pharmacologically active triterpenoid with several potential medicinal properties. There was a correlation between changes of the erythrocyte membrane shape to stomatocytic form and the inhibition of* Plasmodium falciparum* growth caused by a tropical plant* Rinorea ilicifolia* Kuntze lupeol* in vitro* [25]. In addition to indirect activity against* P. falciparum *[25] lupeol isolated from other plants has been reported to inhibit the growth of a several types of bacteria, fungi, and viral species [26–32]. In the present prophylaxis assay the impact of the most active ethyl acetate extract containing lupeol is linear and thus may reflect the indirect effect of lupeol on the erythrocyte membrane. Activity of lupeol from the leaf hexane extract of* Vernonia brasiliana* (L.) Druce (Compositae) against* P. falciparum* has been shown* in vitro* [33]. However, lupeol was found to be inactive* in vivo* when 15 mg/kg was administered per os during four consecutive days to mice infected with* P. berghei*. In our study, two-, four-, and tenfold higher doses of lupeol in the ethyl acetate extract (predominant compound of 27.5%) were administrated per os. The activity implied chemoprotective, indirect activity on the erythrocyte membranes and was not found in the Peters' suppressive test supporting the results by De Almeida Alves et al., 1997 [33]. However, because of the possible effect of other compounds or synergistic effect of several compounds present in the extract the role of lupeol against* P. berghei *remains here unsolved and further studies will be needed in future.

In the present study the* in vivo* assays showed that the studied extracts of* C. macrostachyus* were able to significantly suppress the amount of the parasites in infected Balb/c mice. According to the Tukey's test the suppression of parasitemia was even comparable to the control drug artemether as regards ethyl acetate (500 mg/kg) and isobutanol extracts (down to 100 mg/kg) in Peters' 4-day suppressive test (*p* < 0.001) and ethyl acetate (down to 250 mg/kg) and isobutanol (500 mg/kg) extracts (*p* < 0.001) in the chemoprotective assay.

The most potential antimalarial chemotherapeutic and chemoprotective agent of the studied extracts was the ethyl acetate extract. The isobutanol extract was effective in the suppression of the parasitemia but the highest dose was lethal in the acute toxicity test (500 mg/kg). Thus it can be considered as potential antimalarial drug only in low doses, although all the mice survived in the suppressive and chemoprotective assays. Although the dose-response curves were not very steep, the extracts displayed suppression of parasitemia in a dose-dependent manner. The exceptions were the isobutanol extract in both antimalarial tests and the ethyl acetate extract in the Peters' suppressive test. The effect of isobutanol extract may thus be due to nonspecific activity. In the suppressive test the reason for the relatively high impact of the ethyl acetate extract dose 100 mg/kg compared to the dose of 250 mg/kg remains unknown but may reflect nonspecific activity, which excludes the role of specific compounds like lupeol as the active agents in the suppressive test. These results are in parallel with the previous results by Ziegler et al., 2002 [25], who showed that the effect of lupeol is indirect and is directed to erythrocyte membrane. In the chemoprotective assay the impact of the ethyl acetate extract was linear and may imply the indirect role of lupeol.

## 5. Conclusions

The results obtained in the present study revealed that* C. macrostachyus* stem bark extracts (ethyl acetate, methanol, and isobutanol) have significant antiplasmodial activity against* Plasmodium berghei *both in chemotherapeutic and in chemoprotective way. This upholds folkloric use of the plant and the earlier studies carried out with leaves, root, and fruit. The ethyl acetate extract was the most promising candidate for further drug development. However, the active compounds of the extracts have not been identified, and the antimalarial activity of* C. macrostachyus *may result from a combination of its secondary metabolites. Further testing of the active components of* C. macrostachyus* extracts will be needed to forward* C. macrostachyus* based antimalarial drug development.

## Figures and Tables

**Table 1:** Peters' 4-day suppressive test. Parasitemia (%) in *Plasmodium berghei* inoculated BALB/c mice after treatment with *C. macrostachyus* stem bark extracts.

Animal group/treatment dose (mg/kg)^*∗*^	Parasitemia (%) *Extract*			
	EtOAc	MeOH	H_2_O	isoBuOH
PC	2.15 ± 0.22	0.51 ± 0.23	0.15 ± 0.09	0.00 ± 0.00
500	3.18 ± 0.42	5.84 ± 0.45	5.27 ± 0.38	3.56 ± 0.57
250	7.26 ± 0.31	12.17 ± 0.66	10.44 ± 0.56	7.16 ± 1.28
100	6.64 ± 0.54	13.30 ± 0.67	14.16 ± 0.56	4.97 ± 1.59
NC	17.39 ± 0.27	18.33 ± 0.38	18.72 ± 0.90	18.20 ± 1.30

^*∗*^In each experiment a group of six mice were examined. The values represent the mean and standard deviation; PC = positive artemether control; NC = negative 10% Tween 80 control; EtOAc = ethyl acetate extract; MeOH = methanol extract; H_2_O = aqueous extract; isoBuOH = isobutanol extract.

**Table 2:** Suppression of parasitemia (%) in *Plasmodium berghei* infected BALB/c mice in the Peters' 4-day suppressive test after treatment with *C. macrostachyus* stem bark extracts.

Animal group/treatment dose (mg/kg)^*∗*^	Suppression of parasitemia (%) *Extract*			
	EtOAc	H_2_O	MeOH	isoBuOH
500	82	68	72	80
250	58	34	44	61
100	62	27	24	73
PC	88	97	99	100

^*∗*^In each experiment a group of six mice were examined; PC = positive artemether control; EtOAc = ethyl acetate extract; MeOH = methanol extract; H_2_O = aqueous extract; isoBuOH = isobutanol extract.

**Table 3:** Chemoprotective assay. Parasitemia (%) in the infected mice treated prophylactic with different *C. macrostachyus* extracts against *Plasmodium berghei*.

Animal group/treatment dose (mg/kg)^*∗*^	Parasitemia (%) *Extract*		
	EtOAc	MeOH	isoBuOH
PC	0.20 ± 0.14	0.24 ± 0.17	0.10 ± 0.08
500	0.02 ± 0.02	2.73 ± 0.18	1.33 ± 0.54
250	1.23 ± 0.65	5.66 ± 0.40	2.74 ± 0.27
100	4.51 ± 1.59	6.94 ± 0.18	2.41 ± 0.33
NC	17.36 ± 0.86	16.10 ± 0.66	16.77 ± 0.26

^*∗*^In each experiment a group of six mice were examined. The values represent the mean and standard deviation; PC = positive artemether control; NC = negative 10% Tween 80 control; EtOAc = ethyl acetate extract; MeOH = methanol extract; isoBuOH = isobutanol extract.

**Table 4:** Chemoprotective assay. Suppression of parasitemia (%) in *Plasmodium berghei* infected BALB/c mice after treatment with *C. macrostachyus* stem bark extracts.

Animal group/treatment dose (mg/kg)^*∗*^	Suppression of Parasitemia (%) *Extract*		
	EtOAc	MeOH	isoBuOH
500	100	83	92
250	93	65	84
100	74	57	86
PC	99	99	99

^*∗*^In each experiment a group of six mice were examined; PC = positive artemether control; EtOAc = ethyl acetate extract; MeOH = methanol extract; isoBuOH = isobutanol extract.

## Data Availability

This study was carried out by Jackie K. Obey and is part of her doctoral thesis; all the data are available from her upon request.
